# LncRNA SNHG7 Regulates Gastric Cancer Progression by miR-485-5p

**DOI:** 10.1155/2021/6147962

**Published:** 2021-08-31

**Authors:** Zhongsong Zhao, Xueping Liu

**Affiliations:** Department of Gastroenterology, Shandong Provincial Third Hospital, Cheeloo College of Medicine, Shandong University, Jinan 250031, China

## Abstract

**Background:**

Long noncoding ribonucleic acids (lncRNAs) were closely related to the development of gastric cancer. This study investigated the effect of SNHG7 on gastric cancer progression and its potential molecular mechanism.

**Methods:**

SNHG7 and microRNA-485-5p (miR-485-5p) expressions in gastric cancer tissues and cells were detected by quantitative real-time polymerase chain reaction (qRT-PCR). Cell counting kit-8 (CCK-8), wound healing, and transwell experiments were used to detect cell proliferation, migration, and invasion. The dual luciferase reporter assay, RNA immunoprecipitation (RIP) experiment, and Pearson's correlation analysis were used to confirm the relationship between SNHG7 and miR-485-5p.

**Results:**

SNHG7 expression was increased in human gastric cancer tissues and cells. Knockdown of SNHG7 could notably inhibit the gastric cancer cells proliferation, migration, and invasion. The dual-luciferase reporter assay and RIP experiments proved that miR-485-5p was a direct target of SNHG7. At the same time, further experiments demonstrated that miR-485-5p inhibition reversed the suppression of SNHG7 knockdown on gastric cancer cells proliferation, migration, and invasion.

**Conclusions:**

SNHG7 knockdown could hamper gastric cancer progression via inhibiting miR-485-5p expression, providing a novel understanding for gastric cancer development.

## 1. Introduction

Gastric cancer is one of the most common malignant tumors of the digestive tract [1]. The incidence and death rate of gastric cancer have been decreasing in the past half century, but it is still the second deadliest cancer in the world [2]. Gastric cancer mainly originates from epithelial cells of the gastric mucosa and occurs in the gastric antrum and gastric pylorus [3]. The etiology of gastric cancer is complex, such as genetics, adverse environment, diet, *Helicobacter pylori* (HP) infection, and others [4]. The occurrence and development of gastric cancer are related to multiple factors and genes. Despite the continuous development of surgical techniques, new chemotherapeutic drugs, and a variety of new treatment options [5–8], the current treatment effect for gastric cancer is still limited. Therefore, a deeper understanding of the pathogenesis has great significance for the diagnosis and treatment of gastric cancer patients. At present, lncRNA has become a research focus of antitumor therapy, and its reports in gastric cancer are also increasing.

LncRNA is a class of RNA molecules in which transcripts are more than 200 nucleotides in length and cannot encode proteins [9]. LncRNA can participate in various intracellular signal regulation processes through chromatin modification, transcription activation or interference, and others [10]. Some studies have shown that dysregulation of lncRNA is closely related to gastric cancer invasion, migration, metastasis, prognosis, and et cetera [11, 12]. For example, Xu et al. suggested that ZFAS1 knockdown hampered malignant behavior of gastric cancer cells through the Wnt/*β*-catenin pathway [13]. XIST inhibition suppressed gastric cancer progression and metastasis through regulating miR-101/EZH2 [14]. CCAT2 overexpression accompanied poor prognosis and overall survival for gastric cancer patients [15].

SNHG7 located at 9q34.3 is a potential molecular marker for malignant tumors, such as pancreatic cancer, osteosarcoma, esophageal cancer, bladder cancer, colorectal cancer, and breast cancer, cervical cancer, as well as gastric cancer [16, 17]. Boone et al. reported that SNHG7 involved in the proliferation and apoptosis of breast cancer cells regulated by IGF1 [18]. Zhong et al. found SNHG7 was upregulated, and inhibition of SNHG7 expression could promote cell apoptosis and suppress cell proliferation and invasion in bladder cancer [19]. SNHG7 facilitated cell proliferation and invasion and closely related to poor prognosis in cervical cancer [20]. In esophageal cancer, SNHG7 repressed cell apoptosis and accelerated cell proliferation [21]. These studies indicate that SNHG7 might have certain biological functions in the occurrence, development, and progression of tumor cells. Similarly, SNHG7 also contributed to the progression and development of gastric cancer [22–24]. However, there are few studies on the molecular mechanism of SNHG7 in gastric cancer.

Currently, this study intends to explore the expression and role of SNHG7 and the possible molecule mechanisms in gastric cancer. First, we measured the expression of SNHG7 in gastric cancer. Besides, whether SNHG7 affects gastric cancer cell biological behavior through miR-485-5p was also explored.

## 2. Materials and Methods

### 2.1. Tissues Samples

36 cases of primary gastric cancer specimens were surgically resected and pathologically diagnosed in our hospital from January 2016 to December 2019 were collected, and adjacent tissues were selected as controls. There were 16 males and 20 females with an average age of (51.86 ± 9.38) years, ranging from 32 to 71 years old. The postoperative tissue was quickly stored in liquid nitrogen for subsequent RNA extraction. Inclusion criteria are as follows. (1) All patients were diagnosed with gastric cancer by pathological examination. (2) All patients received surgical treatment and did not undergo related chemotherapy and other related treatment. and (3) Patients with complete clinical data. Exclusion criteria are as follows: (1) Patients with other malignant tumors. (2) Patients with severe liver, kidney, and heart dysfunction and metabolic abnormalities. (3) Patients with previous history of mental illness. and (4) Patients with incomplete clinical data. This study was approved by our hospital Ethics Committee (Approval No. 2015–04). The collection of all specimens was informed by the patients and their families, and informed consent was signed.

### 2.2. Cell Culture

Gastric cancer cells including HS746 T, HGC-27, SNU-1, AGS, and human gastric mucosa epithelial line GES-1 were cultured in the RPMI-1640 medium (Gibco, USA) containing 10% FBS (Gibco, USA) and placed in an incubator with saturated humidity at 37°C and 5% CO_2_. For every 1-2 days, fresh medium was changed. Subculture was performed when the cell fusion reached 80–90%. Logarithmic growth phase cells were taken for subsequent experiments. Each experiment was repeated at least 3 times.

### 2.3. Cell Transfections

Small interfering RNA against SNHG7 (si-SNHG7), si-NC, miR-485-5p mimic (mimic), and inhibitor and their corresponding controls were obtained from Shanghai GenePharma. Cells transfection was performed by Lipofectamine 2000 (Invitrogen, USA). In brief, 5 *μ*l Lipofectamine 2000 was also added into 250 *μ*l serum-free medium. 5 *μ*l si-SNHG7 mimic or controls was added into 250 *μ*l serum-free medium, respectively. Above liquids were mixed and added 1 ml serum-free medium. After 24 h of incubation at 37°C, the cells were harvested for further analysis.

### 2.4. CCK-8 Assay

Cell with 5 × 10^3^ cells/ml was placed in a 96-well plate and incubated for 1, 2, 3, and 4 days, respectively. 10 *μ*l CCK-8 solution (Dojindo, Japan) was added. Each well was measured for absorbance at 450 nm on an enzyme immunosorbent detector.

### 2.5. Wound-Healing Assay

Gastric cancer cells were placed in a 6-well plate. One 200 *μ*l disinfection tip was used to draw a vertical line when the cell fusion degree grew at 90%. The initial distance of scratches (0 h) was measured under a microscope. After 48 h incubation in a constant temperature incubator, the scratch distance was measured and the cell mobility was calculated as described previously [25]. The cell migration rate = (migration distance (0 h) − migration distance (48 h))/migration distance (0 h) × 100%.

### 2.6. Transwell Assay

Cell invasion was assessed by the transwell chamber of 8 mm (Corning, USA) as described previously [26]. Basement membrane matrix (Matrigel, BD, USA) was added to the upper chamber. Cell suspension (1 × 10^5^ cells) was added to the upper chamber. Next, the lower chamber was inserted with 500 *μ*l of RPMI 1640 containing 10% FBS. Cells were incubated for 24 hours in a humidified incubator with 5% CO_2_. Then, the removed cells were fixed and stained. Five fields were randomly selected to count the number of cell invasions.

### 2.7. Dual-Luciferase Reporter Assay

The starBase database (http://starbase.sysu.edu.cn/index.php) was used to predict the binding sites of miR-485-5p and SNHG7. SNHG7-3′UTR fragment containing the binding site and the mutation binding site were inserted into PGL3 vector to construct the wild-type (wt) and mutant-type (mut) vectors of SNHG7. Vectors were cotransfected with miR-485-5p mimic or miR-NC into gastric cancer cells, and cells were cultured for 48 h to detect the luciferase activity of each group.

### 2.8. RIP Assay

RIP experiments were performed using the Magna RIP^TM^ kit (Millipore, USA) as described previously [27]. Cells were lysed by lysis buffer, and the lysate was incubated with magnetic bead-conjugated IgG or Ago2 antibody (Millipore, USA). Coimmunoprecipitated products were collected, and RNA was extracted and purified. QRT-PCR was used to detect the expression of SNHG7 and miR-485-5p.

### 2.9. qRT-PCR

Total RNA was extracted according to the TRIzol method, and cDNA was synthesized by the PrimeScript^TM^ RT Master Mix kit (Takara, Japan). U6 and GAPDH were used as the internal reference. The qRT-PCR amplification reaction was performed in an ABI 7500 instrument (Applied Biosystems, USA) according to the instructions of the SYBR Green PCR detection kit (Takara, Japan). The 2^−ΔΔCt^ method was used to calculate RNAs expression [28]. The primers are given in Table 1.

### 2.10. Statistical analysis

Data were processed using GraphPad Prism5 software and expressed as mean ± SD, and the *t*-test was used for pairwise comparison. Correlation analysis uses Pearson correlation coefficient. *P* < 0.05 was considered statistically significant.

## 3. Results

### 3.1. SNHG7 Expression Is Increased in Gastric Cancer

**Figure 1:**
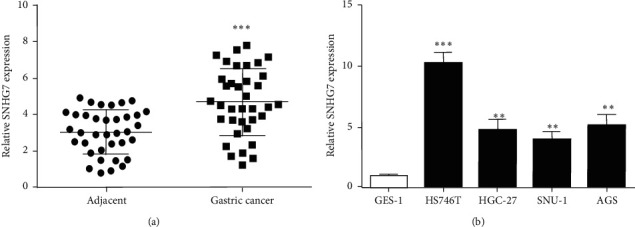
SNHG7's expression detected in gastric cancer tissues and cells. (a) Expression of SNHG7 in gastric cancer tissues. (b) SNHG7 expression elevated in gastric cancer cells. ^*∗∗*^*P* < 0.01. ^*∗∗∗*^*P* < 0.001.

SNHG7 expression was detected in 36 pairs of gastric cancer and adjacent tissues by qRT-PCR. The results revealed that SNHG7 expression in gastric cancer tissues was significantly higher than that of adjacent tissues (Figure 1(a)). To investigate whether there were differences in the expression of SNHG7 between gastric cancer cells and human normal gastric epithelial cells, we tested SNHG7 expression in, HS746 T, HGC-27, SNU-1, and AGS, four gastric cancer cells. Findings suggested that SNHG7 expression was obviously enhanced in all gastric cancer cells versus GES1 (Figure 1(b)). These results suggested that SNHG7 may play an oncogenic role in gastric cancer. It was also found that the expression of SNHG7 in HS746 T cells was higher than that in other gastric cancer cells (Figure 1(b)). Therefore, the HS746 T cell was selected to perform the follow-up experiments.

### 3.2. SNHG7 Knockdown Suppresses Gastric Cancer Cell Proliferation, Migration, and Invasion

**Figure 2:**
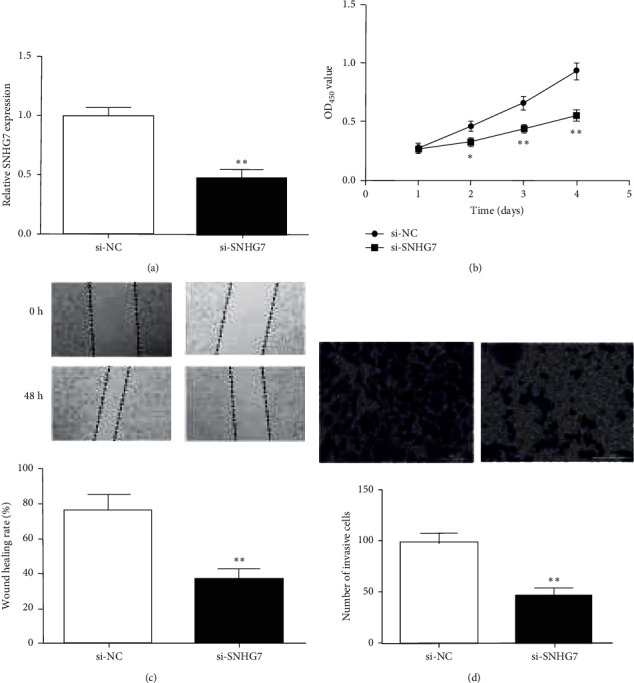
SNHG7 knockdown repressed malignant behavior of HS746 T cells. (a) SNHG7 expression in HS746 T cells after si-SNHG7 transfected. (b) Cell proliferation of HS746 T cells after SNHG7 inhibition. (c) Cell migration rate of HS746 T cells after SNHG7 knockdown. (d) Number of invading HS746 T cells after SNHG7 knockdown. ^*∗*^*P* < 0.05. ^*∗∗*^*P* < 0.01.

The biological effects of SNHG7 in gastric cancer cells were further explored. The results showed that SNHG7 expression was notably decreased by si-SNHG7 (Figure 2(a)), suggesting that si-SNHG7 could successfully inhibit the expression of SNHG7 in HS746 T cells. The CCK-8 assay proved that si-SNHG7 could markedly reduce the absorbance value of HS746 T cells versus the si-NC group (Figure 2(b)). The migration rate of the si-SNHG7 group was also decreased versus si-NC (Figure 2(c)). Similarly, transwell experiments showed that the invasive capacity of si-SNHG7-transfected gastric cancer cells was reduced than that of si-NC cells (Figure 2(d). These results indicated that SNHG7 knockdown could significantly inhibit the proliferation, migration, and invasion ability of gastric cancer cells.

### 3.3. miR-485-5p Directly Binds to SNHG7

**Figure 3:**
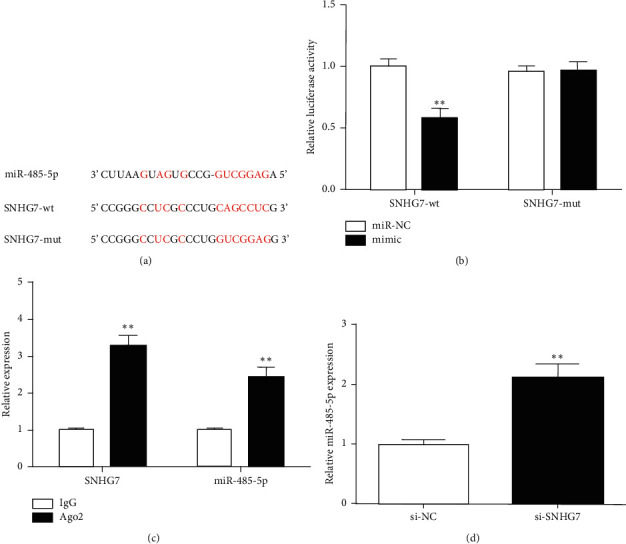
SNHG7 directly targets miR-485-5p. (a) Predicted binding site between miR-485-5p and SNHG7. (b) The luciferase activity in HS746 T cells. (c) SNHG7 and miR-485-5p expressions detected in the RIP assay. (d) miR-485-5p expression in HS746 T cells treated with si-SNHG7. ^*∗∗*^*P* < 0.01.

In order to explore the molecular mechanism of SNHG7 regulating the biological behavior of gastric cancer cells, we predicted miRNAs targeted by SNHG7. We found the complementary binding site of miR-485-5p and SNHG7 (Figure 3(a)). The results of the dual luciferase experiment suggested that in HS746 T cells, miR-485-5p mimic obviously inhibited the luciferase activity in the SNHG7-wt group, while remaining unchanged in cells cotransfected with others (Figure 3(b)). Moreover, expressions of SNHG7 and miR-485-5p were enriched in the Ago2 group (Figure 3(c)). Besides, we detected miR-485-5p expression by the qRT-PCR assay in si-SNHG7-transfected cells. Findings revealed that downregulation of SNHG7 could increase miR-485-5p expression (Figure 3(d)). This suggested that SNHG7 may negatively regulate miR-485-5p expression in HS746 T cells. Since the transfection of SNHG7-mut + mimic had no effect on luciferase activity in HS746 T cells, it was further suggested that their negative regulatory effect was achieved through specific binding in the seed region.

### 3.4. Expression of miR-485-5p in Gastric Cancer

**Figure 4:**
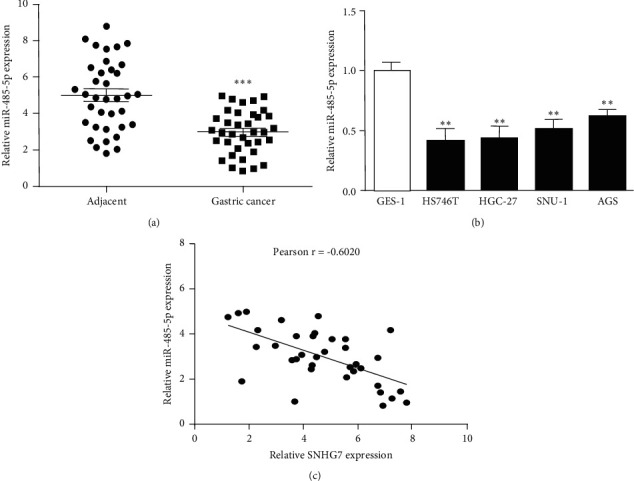
miR-485-5p's expression reduced in gastric cancer. (a) Expression of miR-485-5p decreased in gastric cancer tissue. (b) Expression of miR-485-5p reduced in gastric cancer cells. (c) Correlation analysis between SNHG7 expression and miR-485-5p expression in gastric cancer tissues. ^*∗∗*^*P* < 0.01. ^*∗∗∗*^*P* < 0.001.

In order to confirm miR-485-5p expression in gastric cancer, we used qRT-PCR to detect its expression in gastric cancer tissues and cells. The results showed that compared with adjacent tissues, the expression of miR-485-5p was significantly downregulated in gastric cancer tissues (Figure 4(a)). In addition, miR-485-5p expression levels were obviously decreased in gastric cancer cells versus GES1 cells (Figure 4(b)). After pairing the SNHG7 and miR-485-5p expression data in each sample of gastric cancer tissues, we analyzed whether there is a correlation between their expressions. Interestingly, miR-485-5p expression negatively correlated with SNHG7 expression in gastric cancer tissues (Figure 4(c)).

### 3.5. SNHG7 Facilitates Gastric Cancer Progression via Targeting miR-485-5p

**Figure 5:**
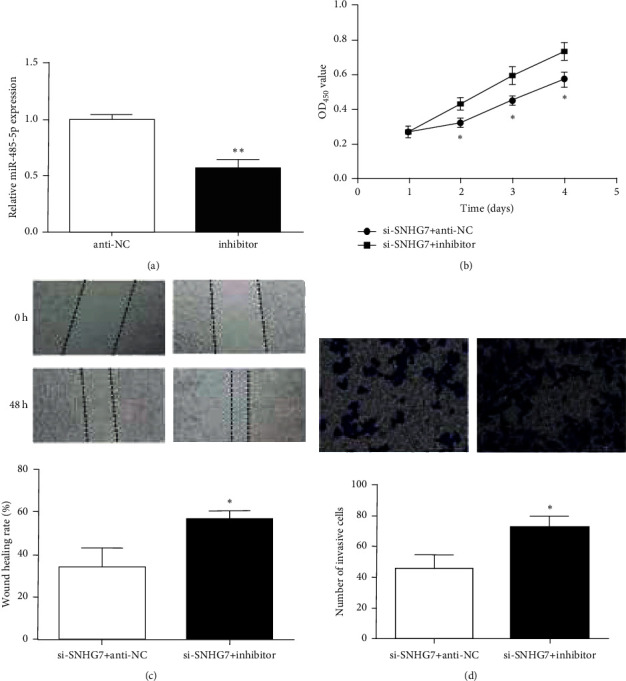
SNHG7 regulated gastric cancer malignant behavior through miR-485-5p. (a) miR-485-5p expression inhibited in HS746 T cells treated with the miR-485-5p inhibitor. (b) Cell proliferation in HS746 T cells transfected with the miR-485-5p inhibitor and si-SNHG7. (c) Cell migration rate in HS746 T cells transfected with the miR-485-5p inhibitor and si-SNHG7. (d) Number of invading HS746 T cells transfected with the miR-485-5p inhibitor and si-SNHG7. ^*∗*^*P* < 0.05. ^*∗∗*^*P* < 0.01.

To further verify whether SNHG7 regulated the malignant behavior of HS746 T cells through miR-485-5p, the inhibitor was transfected into HS746 T cells. Then, qRT-PCR experiments verified that miR-485-5p expression was reduced by the inhibitor (Figure 5(a)). CCK-8 experimental results showed that the inhibitor reversed the effect of SNHG7 knockdown on HS746 T cell proliferation (Figure 5(b)). Moreover, inhibition of miR-485-5p prominently abated SNHG7 silencing-induced cell migration in HS746 T cells (Figure 5(c)). At the same time, the inhibitor attenuated the suppression of si-SNHG7 on cell invasion ability (Figure 5(d)). The above results indicated that SNHG7 regulated the gastric cancer cells malignant behavior by targeting miR-485-5p.

## 4. Discussion

Gastric cancer is a heterogeneous, multifactorial malignant tumor with poor prognosis and difficult to cure [29]. There are no special clinical symptoms in early gastric cancer, most gastric cancer patients are diagnosed as progressive with metastases, and the 5-year survival rate is still low [30]. At present, there is no effective screening method for gastric cancer. In order to prolong the survival time of tumor patients and improve the quality of their life, researchers are encouraged to develop new technologies and methods of cancer treatment. It is necessary to understand the gastric cancer regulatory mechanism at the molecular level. Combined with the currently popular targeted therapy, it will provide important evidence for gastric cancer diagnosis, treatment, and prognosis. This study aimed to provide new biomarkers for the early diagnosis and treatment of gastric cancer by exploring SNHG7 expression and its effect and mechanism in gastric cancer.

SNHG7 was commonly overexpressed in a variety of cancers as an oncogene [17]. Wang et al. found SNHG7 could inhibit P15 and P16 to suppress apoptosis and facilitate the proliferation of gastric cancer cells [22]. Zhang et al. reported that gastric cancer cell migration and invasion were promoted through the miR-34a-Snail-EMT axis by SNHG7 [23]. Here, we conducted a preliminary study on SNHG7 expression in gastric cancer, as well as its functions and regulatory mechanisms. The results found that SNHG7 expression was increased in gastric cancer. After SNHG7 was inhibited in gastric cancer cells, the cell proliferation, migration, and invasion ability were signally repressed. Results suggested that SNHG7 inhibition may hamper the progression of gastric cancer.

A large number of studies have found that miRNAs are related to gastric cancer as tumor suppressor or oncogenic genes [31]. For example, Cao et al. proposed that miR-381 could inhibit TMEM16 A expression to hamper gastric cancer metastasis [32]. Guan et al. reported miR-93 could target TIMP2 to facilitate proliferation and metastasis of gastric cancer [33]. And miR-485-5p was significantly decreased in colorectal cancer (CC), breast cancer (BC), and nonsmall cell lung cancer (NSCLC) and could inhibit the progression of cancers by targeting downstream genes [34–36]. In gastric cancer, miR-485-5p was related to prognosis and overall survival of gastric cancer patients and exerted a suppressor in gastric cancer cell growth and motility by inhibiting NUDT1 [37, 38]. It shows that the antitumor effect of miR-485-5p has biodiversity, which is worthy of further study. Based on previous research, we hypothesized that miR-485-5p plays an important role in gastric cancer progression. Interestingly, miR-485-5p expression was decreased and was inversely related to SNHG7 expression in gastric cancer tissues.

LncRNAs act as a sponge of ceRNA or miRNA molecules by interacting with and inhibiting miRNAs [39]. The bioinformatics analysis revealed that SNHG7 has a complementary nucleotide binding site to miR-485-5p. SNHG7 could specifically target miR-485-5p by the dual-luciferase assay and RIP assay. At the same time, knockdown of SNHG7 in gastric cancer cells was found to increase miR-485-5p expression, indicating that SNHG7 could negatively regulate miR-485-5p. miR-485-5p silencing in cells could partially reverse the inhibition of biological behavior caused by SNHG7 knockdown. Since inhibition of miR-485-5p only partially reversed SNHG7 function, there might be other binding sites or pathways for SNHG7 to participate in tumor promotion.

Since this study was conducted in vitro in cell lines, there are still some deficiencies, including the relatively small number of patients, and the preliminary results may not accurately reflect its expression in gastric cancer, which needs to be repeated in large samples. In addition, how the effect of upregulating SNHG7 or downregulating miR-485-5p on gastric cancer and the results in animals need to be further studied. Although the incidence of gastric cancer has improved in recent years, it still faces tremendous pressure for prevention and treatment due to atypical clinical symptoms in the early stages of gastric cancer. There is an urgent need to discover new molecular markers that provide opportunities for the prevention and control of gastric cancer.

## 5. Conclusion

In summary, expression of SNHG7 was significantly reduced, and SNHG7 inhibition could repress the biological behavior of gastric cancer cells. Rescue experiments confirmed that SNHG7 regulated the gastric cancer cells malignant behavior via the regulation of miR-485-5p. Subsequent research will further explore related mechanisms and signaling pathways on this basis, in order to provide experimental evidence for treatment of gastric cancer.

## Figures and Tables

**Table 1:** Primer sequences for real-time fluorescence quantification PCR.

Gene name	Primer sequences (5′-3′)
GAPDH	F TCCTCTGACTTCAACAGCGACAC
	R CACCCTGTTGCTGTAGCCAAATTC

U6	F CTCGCTTCGGCAGCACA
	R AACGCTTCACGAATTTGCGT

SNHG7	F TTGCTGGCGTCTCGGTTAAT
	R GGAAGTCCATCACAGGCGAA

miR-485-5p	F GGAGAGGCTGGCCGTGAT
	R CAGTGCGTGTCGTGGAGT

## Data Availability

The datasets used and/or analyzed during the present study are available from the corresponding author on reasonable request.
